# The EU’s Habitats Directive Dragonfly *Cordulegaster heros* Theischinger, 1979 in Croatia—Distribution and Habitat Requirements

**DOI:** 10.3390/insects16121284

**Published:** 2025-12-18

**Authors:** Marina Vilenica, Bruno Schmidt, Toni Koren

**Affiliations:** 1 Faculty of Teacher Education, University of Zagreb, Trg Matice Hrvatske 12, 44250 Petrinja, Croatia; 2 Zeleni prsten Public Institution of Zagreb County, 151. samoborske brigade HV 1, 10430 Samobor, Croatia; bruno.schmidt.biol@gmail.com; 3 Association Hyla, Lipovac I 7, 10000 Zagreb, Croatia; koren.toni1@gmail.com; 4 Alexanor, Šipkovica 20a, 10000 Zagreb, Croatia

**Keywords:** Anisoptera, Balkan Goldenring, small lotic habitats, Balkan Peninsula, threats, conservation

## Abstract

*Cordulegaster heros* is an endemic species for Central and Southeastern Europe and one of the species protected under the European Union’s Habitats Directive. To adequately protect this species and its habitats, it is of crucial importance to have detailed information on its distribution, habitat requirements and potential threats to its survival. The main aims of this study were to record *Cordulegaster heros* geographical and altitudinal distribution and habitat requirements (stream width, fine substrate content and habitat shading), along with the importance of protected area network in its conservation and threats to its habitats in Croatia. To achieve those aims, we investigated 201 perennial and intermittent streams across three biogeographical regions (Continental, Alpine, Mediterranean). Additionally, in a small-scale study conducted in streams located within a protected area, we assessed the species’ relationship with water quality. According to the current results, *Cordulegaster heros* was confirmed to reproduce in 44 perennial streams in the Continental and Alpine regions, with a significantly higher number of sites and species’ abundance recorded in the Continental region. As the species was not recorded in the Mediterranean region, its occurrence there remains unverified. The species occurred at an altitudinal range between 150 and 665 m a.s.l., with 77% of the sites being between 150 and 350 m a.s.l. It was mostly documented in streams with widths up to 250 cm, fine sediment content up to 30%, and high habitat shading (>75%). A small-scale assessment of its relationship with water parameters within a protected area revealed a significant correlation with higher concentration of oxygen and lower conductivity, confirming its requirements for clean and well-oxygenated habitats. Approximately 57% of the sites where this species was recorded are within the protected area network. However, because most known occurrences are concentrated within only one area, the Continental region, along with the rather low population densities and anthropogenic threats (e.g., deforestation, hydro-morphological alterations) present at 43% of those streams, further monitoring activities are necessary. The presented results provide a basis for further monitoring of *Cordulegaster heros* and its habitats in Croatia.

## Simple Summary

We investigated *Cordulegaster heros* geographical and altitudinal distribution and habitat requirements, along with the importance of a protected area network in its conservation and threats to its habitats in Croatia. A total of 201 streams were sampled across three biogeographical regions. Additionally, in a small-scale study conducted within a protected area, the species’ relationship with water quality was assessed. *Cordulegaster heros* was recorded at 44 perennial streams. The highest number of streams and species’ abundance were recorded in the Continental region, followed by the Alpine region, while it was absent from the Mediterranean region. It occurred at altitudes between 150 and 665 m a.s.l., preferentially inhabiting streams up to 250 cm wide, with fine sediment substrates up to 30% and dense habitat shading (>75%). A small-scale assessment of its relationship with water parameters revealed a significant correlation with higher concentration of oxygen and lower conductivity, confirming its requirements for clean and well-oxygenated habitats. About 57% of sites where the species was recorded are within the protected area network. However, as most sites are located in the Continental region, population densities of the species are generally low, and anthropogenic threats affect 43% of streams, further monitoring activities are necessary.

## 1. Introduction

Inland waters constitute only 0.01% of water on Earth, yet they have enormous importance for a vast number of organisms, providing home to almost 10% of the extant animal species, as summarized in [1]. They also provide important ecosystem services to humans, such as water, food, and cultural and recreational benefits [2]. Due to the global increase in the human population, freshwater ecosystems and their biota are facing severe threats from water pollution (industrial, urban, agricultural wastewater run off), water abstraction, hydro-morphological alteration (dams, reservoirs, river regulation), the introduction of invasive species, and climate change [1,2,3].

To mitigate and reverse the ongoing global biodiversity loss [4], numerous policies and legal frameworks have been established worldwide [5]. One of the most important ones in Europe is the Habitats Directive (HD), adopted in 1992 [6]. Its main aim is to preserve the most valuable and threatened species and habitats in Europe and ensure their long-term survival by setting out key goals and national measures for their protection [7,8]. However, the HD has been criticized for its biogeographical bias: the most accurate trends originate from Western European countries, while the Southern European fauna is still being highly overlooked, as summarized in [9]. Tang and Visconti [9] hence warned of the need for systematic, standardized, and regular surveys to monitor biodiversity trends, identify drivers of their change, and to determine priority conservation measures.

One of the groups of aquatic macroinvertebrates that are particularly sensitive to habitat alterations are dragonflies (Odonata) [10], which is why they are globally used in assessments of freshwater habitat quality [11]. With their aquatic larvae and aerial (terrestrial) imagines, dragonflies represent an important link in energy and biomass transfer between freshwater and terrestrial habitats [12,13]. They are predators of many other macroinvertebrates, but are also important prey to some vertebrates (amphibians, fish, and birds) [14]. However, dragonflies, like many other insects, face a high risk of extinction due to the combined effects of the aforementioned stressors to freshwater habitats, and 16% of all currently existing dragonfly species face the risk of extinction [15,16]. Additionally, 28% of these threatened species are at risk due to water pollution, while habitat loss from agriculture impacts 61%, logging affects 57%, and urban development influences 29% of these threatened species [15].

As an example, one of the 117 insect species protected under the HD is the dragonfly Balkan Goldenring (*Cordulegaster heros* Theischinger, 1979), listed on the HD’s Annexes II and IV. It is also listed on Annex I of the Convention on the Conservation of European Wildlife and Natural Habitats (Bern Convention) [17]. European Union member states are required to monitor its status, occurrence, and habitats in the different biogeographical regions in which it occurs, and, if necessary, apply conservations measures for its populations and habitats in order to achieve a favorable conservation status [6]. In Croatia, this species has been declared a strictly protected taxon by the Nature Protection Act [18], meaning its habitats should not be modified.

*Cordulegaster heros* is an endemic species of Central and Southeastern Europe [19] that is typically associated with small fast-flowing lotic habitats with low water temperatures (mountain streams, sometimes small rivers) located in forested areas [20]. Many habitats of this species in the Balkan Peninsula are already facing severe threats from climate change, pollution, and various hydrotechnical interventions [21].

In order to be able to monitor and protect the species and its habitats, it is essential to have detailed information on its distribution, habitat requirements, and potential pressures and threats. Knowledge about the distribution of *Cordulegaster heros* in Croatia was until now mostly based on the observation of imagines, e.g., [22,23], while studies focused on larvae or exuviae were rather rare, e.g., [24,25,26].

Considering the lack of detailed data from Croatia and generally from the southern range of distribution of this species, the main goals of this study were as follows: (i) document the geographical and altitudinal distribution of *Cordulegaster heros* in Croatia (based on larval records); (ii) assess the importance of a protected area network in its conservation, (iii) evaluate its habitat requirements, including its association with stream hydrological regimes (perennial vs. intermittent streams), and habitat structure (stream width, percentage of fine sediments, and habitat shading) in Croatian streams; (iv) assess its relationship with water quality in streams within a protected forested area; and (v) document the major threats to its habitats in Croatia.

## 2. Materials and Methods

### 2.1. Study Area

**Figure 1:**
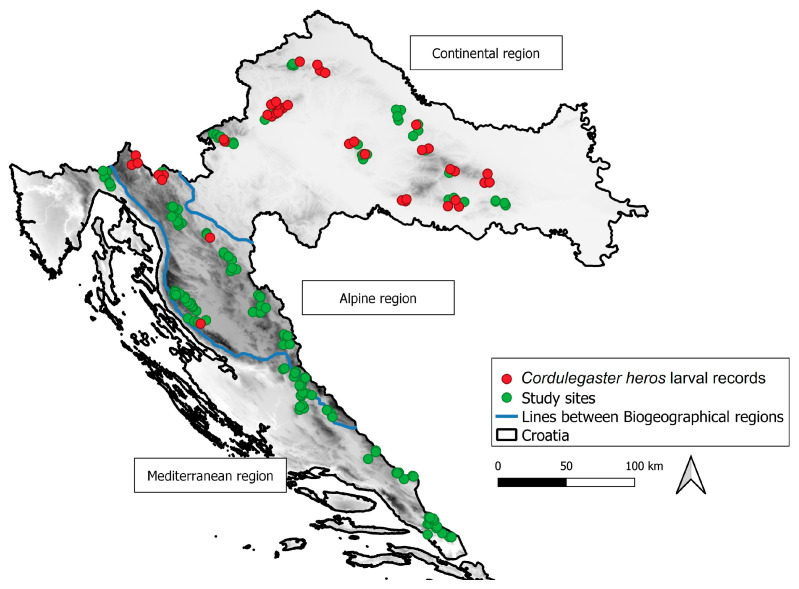
Study sites sampled across three biogeographic regions: green dots—sites without *Cordulegaster heros* records; red dots—sites with *Cordulegaster heros* records (i.e., the species distribution) in Croatia.

The study of *Cordulegaster heros* distribution (geographical and altitudinal) and habitat requirements (association with stream width, percentage of microhabitats with fine sediment substrates and habitat shading) was conducted at 201 sites representing perennial and intermittent small lotic habitats (streams and rivers) in three biogeographic regions (Continental, Alpine, and Mediterranean) in Croatia (Figure 1). Before the fieldwork, study sites were selected in each region using Google Earth digital maps, in line with the species’ habitat choice, as previous records were rather scarce (see Section 4 for more details). Hence, the targeted habitats that were searched for were small streams located in hilly, forested areas, although other types of small streams were also included.

According to Köppen’s classification, the climate in the Continental region of Croatia is temperate humid with warm summers (Cfb), and in the Alpine region it is temperate humid with warm summers (Cfb) and humid boreal (Df), while in the Mediterranean region it is temperate humid with hot summers (Cfa) and Mediterranean with hot (Csa) or warm summers (Csb). In areas with hot summers, the mean air temperature of the warmest month is above 22 °C, while in regions with warm summers, the mean air temperature of the warmest month is lower than 22 °C [27].

A survey of a small-scale species’ relationship with habitat characteristics and physical and chemical water properties was conducted at 15 streams in the Medvednica Nature Park (NP) in the Continental region of Croatia. This area is mostly unaffected by human activities, which is why it is suitable for studying how the species interacts with habitat structure and water chemistry.

Medvednica NP is located on the Medvednica mountain in the north-western part of Croatia, north of the capital city of Zagreb. It extends in a northeast–southwest direction for a length of approximately 42 km covering an area of 17,938 ha. The altitude in the Park ranges from 120 to 1035 m a.s.l. [28]. Medvednica was declared a Nature Park in 1981 due to its high geological, biological [29,30,31,32], and hydrological diversity (with approximately 230 springs and 75 streams) [33]. The largest part (about 83%) of the Medvednica NP is covered with natural forest. The most widespread habitat types are beech forests with large dead nettles, sessile oak and hornbeam forests, and Pannonian beech-fir forests [29]. The Park is located in an area with a moderately warm humid climate with warm summers (mean air temperature of the warmest month is lower than 22 °C) (Köppen climate classification, Cfb) [27]. The average annual precipitation is 1300 mm, while the average annual air temperature at the top of Medvednica is 7 °C [29].

Fieldwork was conducted with all required permits secured (4 May 2022. KLASA: UP7I-352-04/22-08/74, URBROJ: 517-10-1-1-22.3 and 31 May 2023, KLASA: UP/I-352-04/23-08/139URBROJ: 517-10-1-2-23-4).

### 2.2. Sampling and Identification

Sampling was conducted between March 2022 and October 2023. *Cordulegaster heros* larvae were collected from microhabitats with fine substrates (mud, silt, sand) using a benthic hand net (mesh size 0.5 mm) from a surface area of approximately one square meter (m^2^). In case the sampled microhabitat was smaller than one m^2^, abundances (number of individuals) of larvae were recalculated to an area of one m^2^. In most streams across Croatia, a total of 10 replicates (microhabitats) were sampled per stream, while in the Medvednica NP, larvae were counted from the three replicates (microhabitats) at each stream.

Similar habitats in Croatia can also be inhabited by another species of the same genus, *Cordulegaster bidentata* Selys, 184 [19]. The larvae of these two species can be distinguished by the presence of lateral spines on 8th and 9th abdominal segments in *Cordulegaster heros*, which are absent in *Cordulegaster bidentata* [34,35]. After identification, larvae were returned to their habitat.

### 2.3. Environmental Variables

At each stream, we recorded the following habitat characteristics: stream width, percentage of microhabitats with fine substrates, and percentage of shading. In addition, in streams within Medvednica National Park, we measured several physical and chemical water parameters, including water temperature, oxygen saturation and concentration (using the oximeter OXI 96, WTW GmbH, Weilheim, Germany), pH (using the pH-meter 330i, WTWGmbH, Weilheim, Germany), conductivity (using the conductometer Sension 5, Hach, Loveland, CO, USA), water velocity (using a SonTek Flow Tracker, SonTek, San Diego, CA, USA), and water depth (using a handheld meter). Measurements of physical and chemical parameters were taken at three equally spaced points along a transect, running perpendicular to the flow, from the shoreline to the midstream. Furthermore, the main potential threats were identified and assessed at each site.

### 2.4. Data Analysis

Prior to further analyses, data were tested for normality using the Shapiro–Wilk W test. To test differences in habitat characteristics (stream width, fine sediment content, habitat shading) and occurrence of *Cordulegaster heros* among the three biogeographical regions in Croatia, we used Kruskal–Wallis H test with pairwise comparisons of average ranks post hoc test. For differences between the species’ occurrence in perennial and intermittent habitats, Mann–Whitney U Test was used. The Spearman correlation coefficient was used to test the relationship between the species’ abundance and altitude, habitat characteristics (stream width, fine sediment content, habitat shading), and physical and chemical water parameters. All analyses were performed in Statistica 13.0 [36].

To analyze the species’ association with altitude, habitat type (perennial and intermittent), and biogeographical region (Alpine, Continental, Mediterranean), we used data from all sampled streams (N = 201). For intermittent streams that had dried out at the time of sampling, no habitat characteristics were recorded, and thus those streams were omitted from analyses of habitat characteristics, as well as the species‘ association with stream width, fine sediment content, and habitat shading (N = 125). To analyze the species’ relationship with physical and chemical water properties, only the data set collected in the streams of the Medvednica NP was used (N = 45).

Spatial analyses and map creation were carried out using QGIS Desktop version 3.16.3 [37]. The protected area data and biogeographical regions were imported in QGIS as a WFS layer from the Bioportal platform [38], managed by the Croatian Ministry of Environmental Protection and Green Transition. The terrain data was imported as a WFS layer from the State Geodetic Administration [39].

## 3. Results

### 3.1. Cordulegaster heros Distribution and Relationship with Habitat Characteristics in Croatia

**Figure 2:**
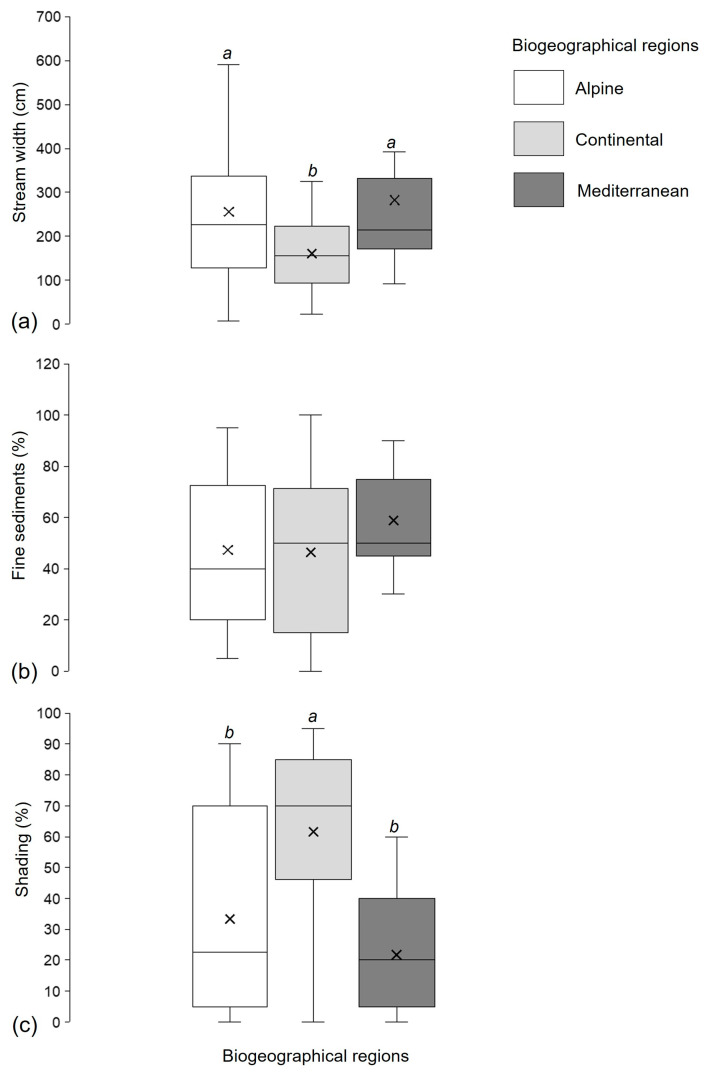
Habitat characteristics in three biogeographical regions in Croatia (Kruskal–Wallis H test): (**a**) stream width (cm), (**b**) fine sediment content (%), and (**c**) habitat shading (%). Different letters (a, b) indicate statistically significant differences among biogeographical regions. Box bottom = Q1 (25th percentile), line inside the box = median (Q2), box top = Q3 (75th percentile), whiskers = minimum and maximum values, and × = mean.

The biogeographical regions significantly differed in stream width (Kruskal–Wallis H test, H (2, N = 125) =16.55, *p* = 0.0003) and habitat shading (H (2, N = 125) = 32.44, *p* < 0.001). The multiple comparisons post hoc test showed that the streams in the Continental region had significantly lower widths (median = 155.5 cm, minimum = 23.5 cm, maximum = 460 cm) and higher habitat shade (median = 70%, minimum = 0%, maximum = 95%) compared to the Alpine (stream width median = 226 cm, minimum = 70 cm %, maximum = 700 cm; shading median = 23%, minimum = 0%, maximum = 90%) and Mediterranean regions (stream width median = 215 cm, minimum = 93 cm %, maximum = 850 cm; shading median = 20%, minimum = 0%, maximum = 60%) (Figure 2a,c). The percentage of fine sediments was comparable among the streams of the three biogeographical regions (H (2, N = 125) = 3.33, *p* = 0.19) (Figure 2b).

**Figure 3:**
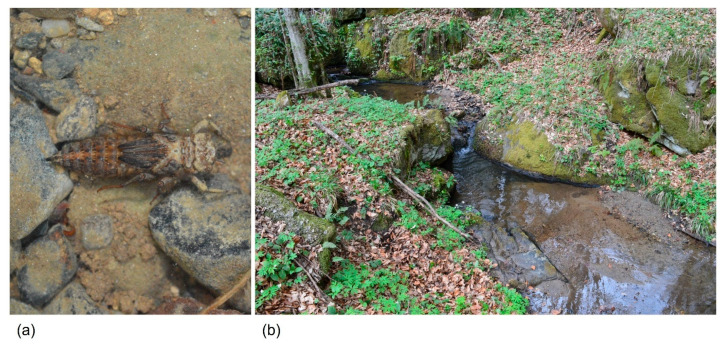
Photo examples of: (**a**) *Cordulegaster heros* larva, and (**b**) its typical habitat (stream in Čazma, Moslavačka gora Regional Park) in Croatia.

**Figure 4:**
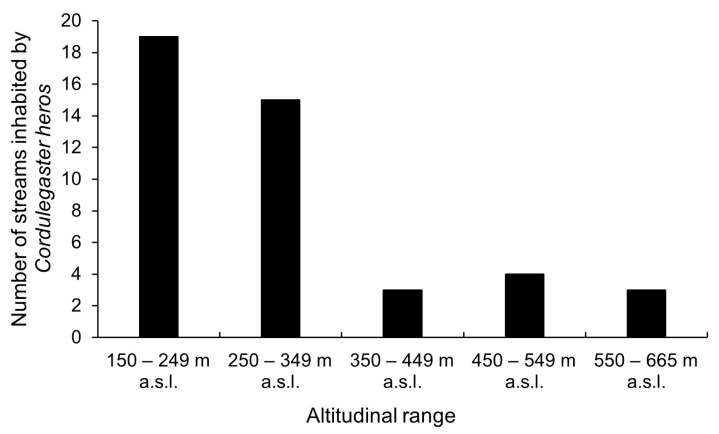
Altitudinal distribution of *Cordulegaster heros* in Croatia.

*Cordulegaster heros* larvae were recorded at 44 (22%) streams studied across Croatia (Figure 1 and Figure 3). The species’ records cover altitudes from 150 to 665 m a.s.l., with significantly higher abundances observed in streams at lower altitudes (Spearman correlation coefficient, N = 201, R = −0.23, *p* = 0.0007). More precisely, 43% of streams where the species was recorded were at altitudes between 150 and 249 m a.s.l., 34% of streams at altitudes between 250 and 349 m a.s.l., 7% between 350 and 449 m a.s.l., 9% between 450 and 549 m a.s.l., and an additional 7% between 550 and 665 m a.s.l. (Figure 4).

**Figure 5:**
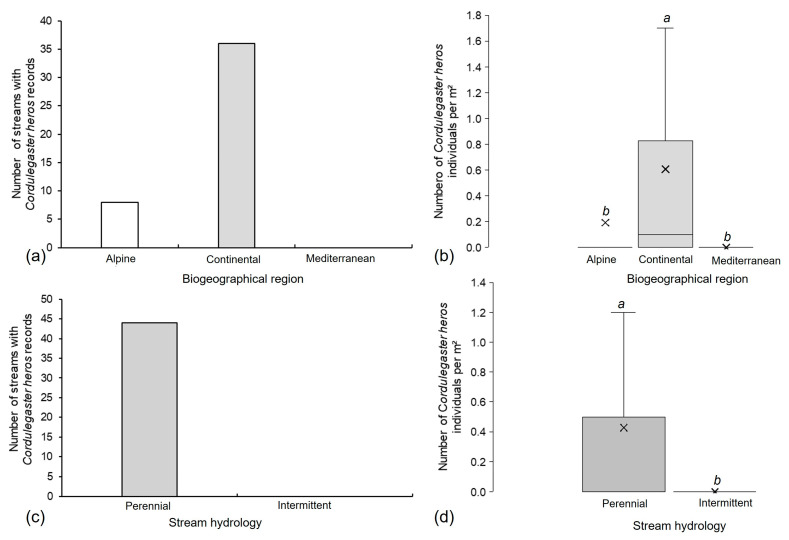
*Cordulegaster heros*: (**a**) distribution and (**b**) abundance (Kruskal–Wallis H test, *p* < 0.05) across biogeographical regions, and its (**c**) association with hydrological regime and (**d**) abundance (Mann–Whitney U test, *p* < 0.05) in perennial (ranging between 0.1 and 8.6 individuals per m^2^) and intermittent streams in Croatia. Different letters (a, b) indicate statistically significant differences among biogeographical regions and between streams with different types of hydrology. (**b**,**d**): box bottom = Q1 (25th percentile), line inside the box = median (Q2), box top = Q3 (75th percentile), whiskers = minimum and maximum values, and × = mean. Different bar colors represent different biogeographical regions (Alpine, Continental, Mediterranean) and stream hydrology (perennial, intermittent).

The highest number of streams where the species was recorded was in the Continental biogeographical region, while it was completely absent from the Mediterranean biogeographical region (Figure 5a). Additionally, a higher abundance was recorded in the Continental region (median = 0.1 individuals per m^2^, minimum = 0 individuals per m^2^, maximum = 8.6 individuals per m^2^), compared to the Alpine region (median = 0 individuals per m^2^, minimum = 0 individuals per m^2^, maximum = 1.9 individuals per m^2^), while no individual was collected in the Mediterranean region (Kruskal–Wallis H test; H (2, N = 201) = 54.03, *p* < 0.001) (Figure 5b). The species occurred exclusively in perennial streams (median = 0 individuals per m^2^, minimum = 0 individuals per m^2^, maximum = 8.6 individuals per m^2^), while no individual was recorded from intermittent habitats (Mann–Whitney U Test; N = 201, U = 3078.00, *p* < 0.001) (Figure 5c,d).

**Figure 6:**
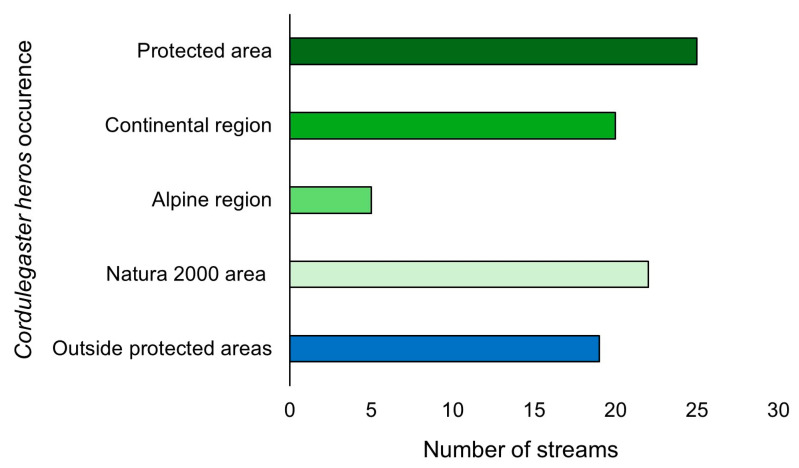
*Cordulegaster heros* occurrence in protected and unprotected areas in Croatia. Green bars show the number of streams in protected areas (total number, numbers in each of the three biogeographical regions, and within Natura 2000 sites). The blue bar shows the number of streams outside protected areas.

A total of 25 sites (57%) where this species occurs are located within the protected area network, out of which 22 (88%) are in Natura 2000 areas. Additionally, 20 of the sites in protected areas (80%) are in the Continental biogeographical region and 5 (20%) are in the Alpine biogeographical region (Figure 6).

### 3.2. Cordulegaster heros Association with Habitat Characteristics in Croatian Streams

**Figure 7:**
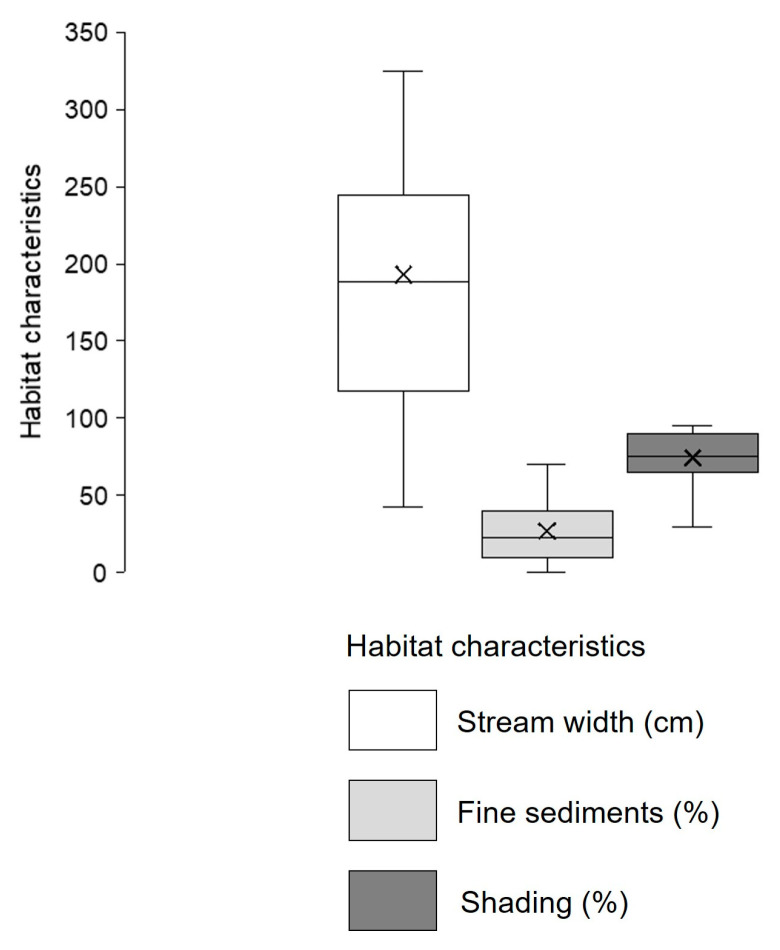
*Cordulegaster heros* habitat characteristics: stream width (cm), fine sediment content (%), and habitat shading (%) in streams in Croatia. Box bottom = Q1 (25th percentile), line inside the box = median (Q2), box top = Q3 (75th percentile), whiskers = minimum and maximum values, and × = mean.

At streams where the species was recorded, the median width was 189 cm, ranging between 43 and 500 cm, the median percentage of fine sediments was 23%, ranging between 5 and 90%, and the median percentage of habitat shading was around 75%, ranging from 30 to 95% (Figure 7).

**Figure 8:**
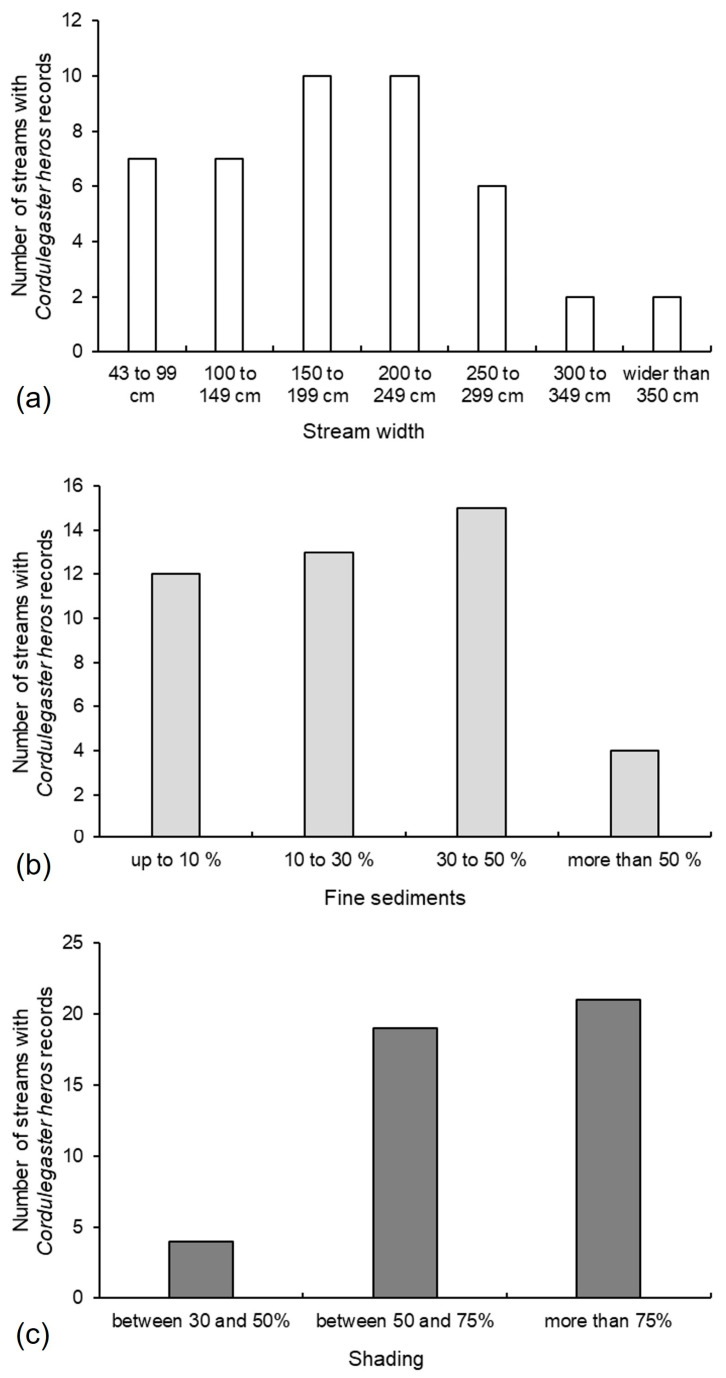
*Cordulegaster heros* habitat characteristics—division and number of streams based on (**a**) stream width (cm) (white bars), (**b**) fine sediment content (%) (light grey bars), and (**c**) habitat shading range (%) (dark grey bars).

Approximately 32% of the streams with species records had widths ranging from 43 to 99 cm and from 100 to 149 cm (16% each). Another 46% fell within the 150–199 cm and 200–249 cm ranges (23% each). Streams measuring 250–299 cm accounted for 14%, while those between 300–349 cm represented 4%. An additional 4% exceeded 350 cm in width (Figure 8a). About 27% of the streams inhabited by the species contained 10% or less fine substrate, 30% had fine substrate ranging from 10 to 30%, 34% had between 30 and 50% fine substrate, and 9% exceeded 50% fine substrate (Figure 8b). A total of 9% of the streams were shaded between 30 and 50%, 43% of streams had a shade range between 50 and 75%, and 48% of streams were shaded more than 75% (Figure 8c).

The abundance of *Cordulegaster heros* larvae was correlated with the lower percentage of microhabitats with fine sediments in their habitat (Spearman correlation coefficient, N = 125, R = −0.50, *p* < 0.001) and higher percentage of habitat shading (R = 0.63, *p* < 0.001). No significant correlations were documented between the species’ abundance and stream width (R = −0.03, *p* = 0.78).

### 3.3. Cordulegaster heros Association with Habitat Characteristics and Physical and Chemical Water Parameters in the Medvednica Nature Park, Croatia

In the Medvednica Nature Park, *Cordulegaster heros* was recorded at 13 out of the 15 studied streams (Table 1). At streams where it was recorded, the median species abundance ranged between one and five individuals per m^2^ (Table 1).

The median stream width ranged from 66 cm in Veliki potok to 257 cm in Trnava, habitat shading ranged between 70% in Pronjak and 95% in Veliki potok and Bliznec, and fine substrate content ranged from 5% in Pronjak, Vidovec, Kraljevac and Bliznec to 40% in Bistra (Table 1).

The median water temperature ranged between 10.5 °C in Rakova noga and 20.3 °C in Vukov potok, the median oxygen saturation ranged from 98.7% in Vukov potok to 106.3% in Veliki potok, and the median concentration of oxygen in water ranged from 8.71 mg/L in Vukov potok to 11.11 mg/L in Markuševački potok. The median value of pH ranged between 7.92 in Dubravica and 8.51 in Rakova noga, conductivity ranged from 205 µS/cm in Vidak to 484 µS/cm in Vukov potok, water velocity ranged from 11 cm/s in Vrapčak, Vidak and Bliznec to 67 cm/s in Dubravica, and water depth ranged between 8 cm in Mali potok and Bliznec and 28 cm in Rijeka (Table 2).

The abundance of *Cordulegaster heros* in Medvednica Nature Park was correlated with higher habitat shade (Spearman correlation coefficient, N = 45, R = 0.37, *p* = 0.01), a higher concentration of oxygen in water (R = 0.39, *p* = 0.009) and lower conductivity (R = −0.50, *p* = 0.0004). Correlations between the species abundance and stream width (R = −0.01, *p* = 0.96), fine substrate content (R = 0.28, *p* = 0.06), water temperature (R = −0.27, *p* = 0.07), oxygen saturation (R = 0.23, *p* = 0.14), pH (R = −0.01, *p* = 0.96), water velocity (R = −0.27, *p* = 0.07), and water depth (R = −0.19, *p* = 0.20) were not significant.

### 3.4. Threats to Habitats and Populations of Cordulegaster heros in Croatia

**Figure 9:**
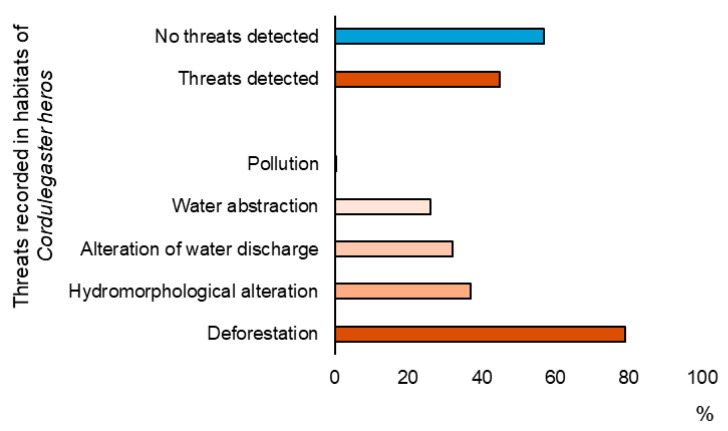
Anthropogenic pressures recorded at streams where larvae of *Cordulegaster heros* were documented in Croatia. Blue bars represent the number of streams without anthropogenic pressures; orange bars show the number of streams affected by anthropogenic threats, including both the total number and separate counts for each specific threat observed.

**Figure 10:**
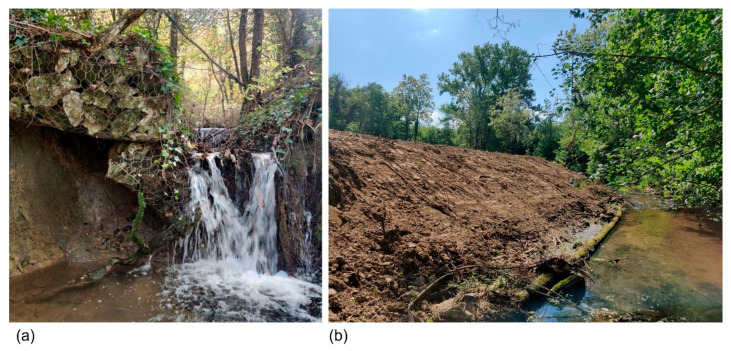
Examples of anthropogenic pressures: (**a**) hydromorphological alterations (small dams) (Ivanšćica, Gačice, Bednja River tributary); (**b**) deforestation (Petrovina, Lukavac) observed at studied streams in Croatia.

Habitat degradation was documented in 19 (43%) of the 44 streams where the species was recorded, attributed to one or more anthropogenic factors (Figure 9). The most common anthropogenic pressure was deforestation, affecting 79% of the impacted streams, followed by hydromorphological changes (e.g., small dams, downstream channelization) impacting 37% of the affected streams, alteration of water discharge (e.g., due to the effect of the global climate change extremes) influencing 32% of affected streams, water abstraction (e.g., for irrigation purposes) affecting 26% of impacted streams, and water pollution (e.g., domestic waste disposal) observed at 1 (0.05%) of the impacted streams (Figure 9 and Figure 10).

## 4. Discussion

### 4.1. Cordulegaster heros Prefers Perennial Streams in Submontane Areas of Continental Biogeographical Region in Croatia

*Cordulegaster heros* is an endemic species in Central and Southeastern Europe. Due to its specific habitat requirements, its distribution is dispersed (localized), spreading from the Italian Slovenian border eastwards to Ukraine and Romania, southwards to Bulgaria, and northwards to the Czech Republic and Slovakia [40,41]. Similarly, a localized pattern was also observed in Croatia, with the species being highly localized in perennial streams in montane and submontane areas, predominantly in the Continental biogeographic region at altitudes between 140 and 350 m a.s.l., in line with its known biogeographic and altitudinal range, as summarized in [42,43]. On the other hand, despite the substantial research effort used, it was rather rare in the Alpine region and completely absent from the Mediterranean region and intermittent habitats, most probably due to the lower habitat suitability. More precisely, the streams in those two regions were often wider (more than 250 cm) and less shaded (30% or less) than those in the Continental region (median width 155 cm, and shade 70%), where streams were more in line with the known species requirements (approximately one meter wide, mostly shaded habitat) [43,44].

Previous studies mentioned this species in the Mediterranean region in lotic habitats near the Knin town [22,23]. However, since only imagines were reported, its occurrence in the larval stage in the Mediterranean region still needs to be confirmed. The observed imagines could possibly originate from the southern Bosnia and Herzegovina, where the species is widespread [45]. Overall, its absence from the Mediterranean region in Croatia and general rarity in the Mediterranean region of the Balkan Peninsula could be explained by its habitat requirements, as it is most commonly encountered in perennial clean forest streams [41]. Such habitats are shaded and characterized by cold, well-oxygenated water and stable water flow e.g., [24]. On the contrary, streams in the Mediterranean region are often characterized by lower shade, a higher water temperature, the rarity or lack of soft substrates and, most importantly, seasonal droughts [23,46]. Additionally, in the Mediterranean region, forests are mostly degraded by deforestation, agriculture, and fires [47], further decreasing the suitable habitats for the species. In Central Europe, *Cordulegaster heros* larvae were documented in intermittent streams [48], where they cope with drying events using burrowing or escaping strategies [40]. Those studies associated higher larval survival with a greater share of fine substrate material in their habitats. However, the authors suggested the species has the ability to tolerate short-term drying by burrowing into fine moist sediment, while prolonged drying events most probably limit its occurrence in intermittent habitats [40,48]. The presence of fine sediments that remain moist during the drying phase in intermittent streams is crucial for survival of many dragonflies [23], including *Cordulegaster heros* [40,48]. Hence, despite the species’ potential to inhabit intermittent streams, the combination of the aforementioned conditions in the Mediterranean streams most probably resulted in such habitats being unsuitable for life cycle completion there.

Prior to this research, several studies have reported the species’ occurrence in Croatia [22,23,25,26], where larvae were recorded in a total of 11 streams (3 streams in the Banovina region [25]) and an additional 8 streams in the Papuk Nature Park [26]). Additionally, Ergović et al. [24] reported the species in its larval stage from the Pannonian Lowland Mountains, Medvednica, Papuk, and Psunj; however, no detailed sites were provided. Hence, with our study, the current number of known streams where the completion of the species’ life cycle was confirmed rises to 55. Although Croatia lies in the center of its distribution range, our results show that it is not as common as in neighboring Slovenia, where it was documented in more than 200 streams [42]. However, such a high presence of the species in Slovenia compared to Croatia is most probably a result of higher habitat availability in Slovenia, i.e., a higher number of forested streams in hilly areas that can be inhabited by this species.

### 4.2. Cordulegaster heros Prefers Clean Well-Oxygenated Forested Streams with High Habitat Shade

*Cordulegaster heros* was exclusively found at its main habitat type, (sub)montane streams [41], although some studies have documented its presence in drainage channels with fast water flow along forest edges, as well as in karst springs in the southern part of its range [19,49]. It was documented in streams with a wide range of widths, from 43 cm to as high as 500 cm, which is most probably the reason why it did not show significant correlations with stream width. However, the highest number of habitats where the species occurs in Croatia are up to 250 cm wide. Previous studies have indicated that the optimal stream width for this species is approximately one meter [43,44], though it has also frequently been observed in streams with bed widths ranging between 50 cm and 350 cm [50].

Although *Cordulegaster heros* inhabits shaded streams characterized by fast water currents, its larvae can most commonly be found in calmer marginal areas, such as pools along the stream edge, where they occupy microhabitats with a reduced flow velocity (approx. 2.6 cm/s), shallower water depth (approx. 5.6 cm), and a sandy substrate covered with a thin layer of organic silt and leaves [45,51]. In our study, about 27% of the streams inhabited by this species contained approximately 10% fine substrates, with an additional 30% of streams exhibiting fine substrate proportions between 10 and 30%, which can be considered as rather low considering the species’ microhabitat choices. In line with such habitat characteristics, the species was highly associated with habitats with a lower fine substrate content. Such results may reflect its characteristic occurrence in heavily shaded habitats, as most of the studied streams with a closed canopy also had a lower share of fine sediment content (approximately 30%). Our findings confirmed the results of previous studies suggesting that habitat shade of about 80% is optimal for this species [50,52]. A similarly high association between *Cordulegaster heros* abundance and tree coverage was documented in a smaller scale part of the study that was conducted in the protected forest area (Medvednica NP). Furthermore, in this sub-set of streams, the species was highly associated with streams characterized by a higher concentration of oxygen in the water and lower conductivity, confirming its affinity for clean and well-oxygenated streams [20,50]. No significant correlation was documented between its abundance and water temperature, most probably as water temperature in streams in the Medvednica Mountains ranged between 10.7 °C in and 20.7 °C, which is in line with optimal thermal conditions of this species [50,53].

### 4.3. Threats to Cordulegaster heros’ Habitats and Implications for Its Conservation

Due to better knowledge about *Cordulegaster heros* distribution in Europe, the most recent IUCN assessment changed its European conservation status from near-threatened to least concern [54]. A total of 55 streams in Croatia where this species completes its life cycle (current and literature data combined) represents a moderately good conservation status as the highest number of streams are localized in the Continental biogeographical region. Such localization increases the risk from climate and land-use pressures, such as local disasters (fires, droughts, pollution), which could deteriorate habitat conditions or lead to the habitat’s low(er) suitability for survival of this habitat specialist. For comparison, this species was recorded at more than 60 sites in Slovakia (the northern border of its occurrence range in Europe) [55,56,57], and at more than 200 sites in Slovenia [42]. Additionally, at streams where it was recorded, the species was present with a median abundance of 0.8 individuals per square meter of the habitat, ranging between 0.1 and 8.6 individuals per square meter, similar to populations in Czechia [43]. On the other hand, some populations in Slovakia were much more abundant (up to 25 individuals per 0.25 square meters) see in [55]. However, despite the fact that most of the sites where the species occurs in Croatia are in only one biogeographical region (Continental), and despite its generally rather low population densities, approximately 57% of the *Cordulegaster heros* habitats are within the protected area network, mostly as Natura 2000 sites, indicating that they are under a high level of conservation activities under EU legislation. However, many of the sites, even those in protected areas, still lack active habitat protection, as anthropogenic threats were documented at 43% of the streams, with the highest occurrence of deforestation, impacting 79% of the threatened streams, and hydromorphological alterations (e.g., small dams, downstream channelization) impacting 37% of the affected streams.

Hence, within the species’ areal, the main current pressures and threats are primarily related to both the aquatic (stream) and the surrounding terrestrial (forest) habitats [41,44]. Climate change has already resulted in changes in the hydrological regime of numerous lotic habitats in the Balkan Peninsula, where some of the habitats have changed their hydrological regime from perennial to intermittent, while some have completely dried up [46,58]. Many streams are significantly affected by various hydrotechnical interventions (e.g., spring captation, channelized stream beds, deepened, narrowed bottom and riparian zone, removed riparian vegetation, logging) and pollution (e.g., wastewater disposal from agriculture, industry, urban areas) [46].

Since the species’ long-term survival is related to the quality of its habitats and the availability of suitable microhabitats [20], it is mandatory to reduce existing threats and to prevent further negative anthropogenic influences, such as hydromorphological alterations, water extraction, pollution, and deforestation. Additionally, it is necessary to continuously monitor the species’ population densities in both the Alpine and Continental biogeographical regions in order to detect any negative trends, which would merit more significant conservation measures. Additionally, we recommend implementing conservation actions such as the improvement of habitat management in protected areas (through forestry regulation and maintenance of the riparian forest canopy), inclusion of additional sites into the Natura 2000 network (e.g., habitat with the highest species population, stream in Čazma), monitoring of habitat characteristics and hydrology, and maintaining a good ecological status for the stream corridors between the populations located at different mountains in Croatia (e.g., Pannonian lowland mountains Medvednica, Papuk, Psunj).

## 5. Conclusions

Here we provide detailed insights into the geographical and altitudinal distribution and habitat requirements of *Cordulegaster heros* in Croatia, as well as the anthropogenic pressures present at its habitats. With the literature data, the number of streams where the species occurs in Croatia is 55. Since all its habitats are perennial, and predominantly located in the Continental biogeographical region, future monitoring is essential, especially considering increasing deforestation and climate change, which alter and decrease habitat suitability for this species. Our results provide a basis for further monitoring of *Cordulegaster heros* and its habitats in Croatia, including future assessments of its geographical range, habitat quality, threats, and population size and trends. Additionally, the frequent co-occurrence of this species with *Cordulegaster bidentata*, *Onychogomphus forcipatus* (Linnaeus, 1758), and *Calopteryx virgo* (Linnaeus, 1758) [19] means that the habitat protection measures implemented for *Cordulegaster heros* will also provide conservation benefits to these syntopic species.

## Figures and Tables

**Table 1:** Abundances of *Cordulegaster heros* (N) and habitat characteristics (altitude, stream width, shading, fine substrate content) in streams of the Medvednica Nature Park, Croatia. Species abundance (number of individuals per square meter (ind/m^2^)) and stream width are shown as median with minimum and maximum values in brackets.

Streams	N (ind/m^2^)	Altitude (m a.s.l.)	Stream Width (cm)	Shading (%)	Fine Substrate (%)
Trnava	3 (2–4)	340	257 (241–263)	90	10
Markuševački potok	2 (2–3)	320	224 (197–226)	90	35
Rakova noga	1. (1–2)	517	188 (173–207)	80	10
Vidak	3 (2–4)	314	135 (121–148)	85	20
Pronjak	1 (0–1)	282	177 (176–191)	70	5
Bidrovec	0 (0–0)	351	190 (2170–210)	90	15
Vidovec	1 (0−2)	336	160 (150–170)	80	5
Veliki potok	2 (0–2)	310	66 (54–74)	95	10
Vrapčak	1 (0–1)	339	113 (98–122)	75	10
Bliznec	4 (2–5)	393	105 (90–120)	95	5
Bistra	5 (4.0–6)	280	160 (140–170)	90	40
Rijeka	0 (0–1)	260	201 (198–252)	80	15
Vukov potok	0 (0–1)	314	154 (121–178)	90	20
Dubravica	0 (0–0)	226	67 (56–71)	90	10
Kraljevac	0 (0–0)	567	216 (198–227)	80	5

**Table 2:** Physical and chemical water parameters (shown as median with minimum and maximum values in brackets) in streams of the Medvednica Nature Park, Croatia.

Streams	Water Temperature (°C)	Oxygen Saturation (%)	Oxygen Concentration (mg/L)	pH	Conductivity (µS/cm)	Water Velocity (cm/s)	Water Depth (cm)
Trnava	10.8 (10.8–11.2)	102.8 (102.7–103.1)	11.09 (11.01–11.15)	8.38 (8.35–8.39)	255 (253–257)	18 (6–32)	12 (9–17)
Markuševački potok	10.6 (10.6–10.8)	103.1 (103.0–103.1)	11.11 (11.08–11.15)	8.32 (8.30–8.46)	266 (266–267)	58 (8–88)	8 (7–10)
Rakova noga	10.5 (10.5–10.6)	103.6 (103.4–103.6)	11.01 (10.08–11.06)	8.51 (8.49–8.53)	337 (336–337)	441 (11–69)	12 (4–15)
Vidak	12.3 (12.3–12.5)	103.3 (103.3–103.4)	10.79 (10.75–10.79)	8.05 (8.04–8.11)	205 (204–205)	11 (5–41)	13 (7–24)
Pronjak	12.3 (12.3–12.4)	100.7 (100.7–101.0)	10.56 (10. 46–10.59)	8.23 (8.03–8.26)	247 (246–247)	22 (5–44)	10 (4–12)
Bidrovec	13.9 (13.7–14.6)	105.0 (104.5–105.4)	10.50 (10.28–10.50)	8.44 (8.36–8.45)	267 (267–268)	33 (8–61)	18 (10–26)
Vidovec	15.1 (15.0–15.5)	106.0 (105.4–106.3)	10.32 (10.16–10.37)	8.42 (8.40–8.46)	385 (382–386)	38 (11–41)	13 (6–18)
Veliki potok	14.5 (14.3–14.7)	106.3 (106.3–106.6)	10.59 (10.53–10.65)	8.36 (8.34–8.37)	351 (349–352)	38 (11–41)	12 (6–16)
Vrapčak	15.2 (15.1–15.4)	105.9 (105.6–106.0)	10.29 (0.10.29–10.34)	8.47 (8.47–8.49)	437 (434–437)	11 (2–13)	9 (6–13)
Bliznec	17.1 (17.0–17.4)	106.0 (106.0–106.1)	9.80 (9.73–9.83)	8.29 (8.28–8.31)	405 (404–405)	11 (2–24)	8 (4–10)
Bistra	14.7 (014.7–15.0)	105.8 (105.4–105.8)	10.42 (10.34–10.44)	8.21 (8.10–8.24)	225 (222–225)	16 (5–27)	9 (8–17)
Rijeka	15.7 (15.5–15.9)	104.3 (103.9–104.6)	10.09 (9.99–10.16)	8.17 (8.16–8.20)	308 (307–308)	16 (2–63)	28 (7–36)
Vukov potok	20.3 (20.1–21.8)	98.7 (98.4–99.6)	8.71 (8.44–8.85)	8.35 (08.34–8.35)	484 (482–484)	16 (9–21)	13 (6–26)
Dubravica	12.4 (12.4–12.8)	100.3 (100.0–100.7)	10.55 (10.45–10.60)	7.92 (7.90–7.93)	480 (474–481)	67 (21–83)	13 (4–27)
Kraljevac	16.9 (16.5–17.1)	103.4 (103.4–103.8)	9.43 (9.40–9.55)	8.25 (8.24–8.26)	393 (393–394)	22 (2–72)	15 (11–18)

## Data Availability

The original contributions presented in this study are included in the article. Further inquiries can be directed to the corresponding author.
